# Welfare assessment traits, milk quantity and quality, and profitability of Anatolian buffalo cows confined in closed-tied or semi-open free-stall barns can be affected by supplementary feeding at milking

**DOI:** 10.5713/ab.23.0366

**Published:** 2024-01-20

**Authors:** İbrahim Cihangir Okuyucu, Ahmet Akdağ, Hüseyin Erdem, Canan Kop-Bozbay, Samet Hasan Abacı, Ali Vaiz Garipoğlu, Esin Hazneci, Nuh Ocak

**Affiliations:** 1 Department of Animal Science, Faculty of Agriculture, Ondokuz Mayis University, 55139, Samsun, Türkiye; 2 Department of Animal Science, Faculty of Agriculture, Eskisehir Osmangazi University, 26480, Eskisehir, Türkiye; 3 Department of Agricultural Economics, Faculty of Agriculture, Ondokuz Mayis University, 55139, Samsun, Türkiye

**Keywords:** Barn Condition, Feeding Management, Milk Production, Partial Budget Analysis, Temperament Score, Water Buffalo

## Abstract

**Objective:**

This study was conducted to evaluate the effect of offering (OSF) or not (NSF) supplemental feed at milking on temperament (TS), udder hygiene (UHS) and body condition (BCS) scores, and milk yield per milking (MYM), milk quality traits, and profitability of primiparous Anatolian buffalo cows at 90 days of lactation confined in closed-tied (CB) or semi-open free-stall (OB) barns.

**Methods:**

In Experiment I, 108 cows were selected to encompass four treatments (OB-OSF, OB-NSF, CB-OSF, and CB-NSF) of 27 cows, considering barn type (OB and CB) and supplementary feed (OSF and NSF) at milking. In Experiment II, 60 OB cows were selected to encompass one of five groups of 12 cows each: i) no supplemental feed (CON), ii) commercial concentrate (CC), iii) CC + corn silage (CCS), iv) CCS + alfalfa hay (CSA), or v) CC + ryegrass silage (CRS) at milking.

**Results:**

The TS and UHS of the OB and OSF cows were lower (better) than those of the CB and NSF cows, respectively. The OSF increased milk protein, lactose, and solids-not-fat but decreased milk freezing point and electrical conductivity compared with the NSF. The MYM and milk fat of the OB-OSF cows were higher than those of the OB-NSF and CB-NSF cows. The TS and UHS of the cows negatively correlated with MYM and some milk chemicals (fat, protein, and solids-not-fat), but BCS correlated positively. The TS and milk electrical conductivity of the CCS, CSA, and CRS cows were lower than those of the CON and CC cows, but BCS, MYM, and milk fat were higher. Partial budget analysis identified a higher net profit for supplemental feed-offered groups (OB-OSF, CCS, CSA, and CRS).

**Conclusion:**

Offering roughage with concentrates at milking for indoor primiparous buffalo cows is more conducive to well-being, milk yield, milk quality, and economy.

## INTRODUCTION

In some countries, including Türkiye, lactating water buffaloes (*Bubalus bubalis*) are confined in closed-tied stalls or set loose in semi-open free-stall barns with the same modern systems used for dairy cows during part or all of the year [1–3]. This system, in which the water buffalo cows (hereafter buffalo) are confined and fed a balanced diet in barns and milked mechanically once or twice a day, aims to increase the productivity indices of buffalo cows [3–5]. Nevertheless, this case not only represents a fundamental challenge for the dairy buffalo sector in terms of sustainable milk yield and quality traits as well as profitability [6] but also leads to managerial issues and consumer sensitivity regarding animal welfare and food safety [2,7]. When buffaloes move away from their natural habitats, housing systems, feeding, handling, stockperson-animal interaction, and machine milking pose economic and well-being challenges for the buffalo industry [4,5,7].

The welfare criterion is a principal concept that must be considered in developing buffalo production systems because animal welfare is a critical determinant factor for food quantity and quality for producers and consumers [3,4,8]. Indeed, balancing milking welfare and productivity is essential because it increases productivity while reducing feed and labor costs [1]. As such, some researchers [3,8,9] have focused on how the milking, feeding, and housing practices affect some welfare assessment traits such as temperament (TS), udder hygiene (UHS), and body condition (BCS) scores and as a result, productivity (e.g., milk yield and quality) of buffaloes. A few studies have evaluated the mutual relationships between these welfare assessment traits [3,9,10] and milk yield and quality traits [8,9,11], including freezing point (FP) and electrical conductivity (EC) [12]. These studies have indicated that milk yield and some milk features of lactating buffalo cows enhanced as the welfare indicators related to barn conditions and practices of milking and feeding improved. Also, these studies have reported that animal welfare and actual state are more closely linked to animal-based welfare indicators than resource-based indicators [13], regardless of housing or management.

Supplementary feeding and offering supplemental feeds (hereafter feeds) at milking for buffalo cows confined in different barn conditions may be beneficial because it promotes calmness and allows them to become accustomed to the milking procedures. Nonetheless, only a few studies [3,14,15] on barn conditions and milking management in buffaloes have focused on the association of these welfare criteria with milk yield, chemical composition, and physical properties, which are crucial to the dairy buffalo industry [2,16]. Indeed, for indoor buffaloes, there is a gap in the literature on how the barn type (BT) and supplementary feeding (SF) interaction at the milking influence the environment- and animal-based welfare assessment criteria [13], milk quantity and quality, and economic returns. In this context, we aimed i) to evaluate the effects of SF at milking on subjectively scored welfare assessment traits (TS, UHS, and BCS) and milk yield per milking (MYM), milk components such as fat, protein, lactose, solids-not-fat (SNF), and minerals, and physical traits such as milk density, FP, and EC of primiparous buffalo cows confined in semi-open free-stall (OB) or closed-tied stalls (CB) barns, ii) to assay the association between these variables, and iii) to investigate changes, if any, in the welfare assessment traits, the MYM and milk quality traits, and economic returns as influenced by different feeds offered at milking.

## MATERIALS AND METHODS

### Animal care and farms recruited

This study, designed as two observational experiments, was performed on Anatolian buffalo cows kept under zero grazing in closed or semi-open barn conditions. Before the experiments began (October 2022), the Ethical Committee of Ondokuz Mayis University for Experimental Animals confirmed that ethics committee approval was not required for this study under Permit No 2022/45. This committee also determined that these experiments were not an unnecessary repetition of previous studies. Therefore, the study procedure, animal handling, and welfare protocol were performed based on the guidelines for the ethical use of animals for experimental and other scientific purposes.

Farms were enrolled to be representative of the barn conditions and milking management (SF at milking) of the study based on the statement of the owner farmer to gather information on i) characteristics of the cows, ii) management, iii) general herd health, iv) milking, and v) diet. As a consequence, farms that i) had closed or semi-open BT and ii) portable milking machines, iii) supplied continuously experimental feed components (total mixed ration, TMR or partial mixed ration, PMR), and iv) measured milk yield were recruited. Furthermore, on enrolled farms, cows were fed a TMR twice daily before and after milking at the trough in front of stalls and had free access to drinking water. The same TMR on some farms with closed or semi-open BT was offered at milking. In contrast, commercial concentrate or different roughage (corn silage, alfalfa hay, and ryegrass silage) with concentrate (hereafter PMR, unless otherwise stated) were offered at milking on others with semi-open BT.

The TMR used at both milking and non-milking times had a forage: concentrate ratio of 60:40 (on dry matter [DM] basis) composed of the same ingredients (grass and alfalfa hay and maize silage, concentrates, and vitamin and mineral supplements). The mean content of the TMR for metabolizable energy (ME), crude protein (CP), neutral detergent fiber (NDF), and acid detergent fiber (ADF) was 11.3 MJ, 140 g, 340 g, and 200 g per kg DM. The ME value and the CP, NDF, and ADF contents of the commercial concentrate were 11.7 MJ ME, 170, 280, and 150 g per kg DM, respectively. In contrast, corresponding values of different PMRs were about 10.4 MJ ME, 170, 360, and 210 g per kg DM, respectively.

In all farms, stockperson, animal management, and milking routines were not changed. The milking processes include allowing calves to suckle their dams for less than one minute immediately before milking, keeping calves close to their dams until the end of milking, and ensuring that calves suckle the residual milk after milking. Additionally, these processes included the behavior of the stockperson during the milking and preparation of the cows (such as teat washing and drying). Also, the cows were not subjected to any pre- and/or post-milking practices.

### Animals, study design, and supplemental feed

#### Experiment I

Based on BT (OB or CB) and SF practice (offered SF at milking, OSF or not offered SF, NSF) at milking, farms were recruited in Experiment I. On four farms recruited in Experiment I, 200 cows (body weight of 450±30 kg) at 60±15 days in milk were observed for an average of 30 days for heat and diseases (endometritis, mastitis, claw disorders, fever, ketosis, and displaced abomasum). After all, based on the availability of clinically healthy cows at approximately 90 days in milk, 108 primiparous cows were selected to evaluate the effect of SF (OSF or NSF) at milking on the welfare assessment traits, milk quantity and quality, and profitability of the cows at two barn conditions (OB or CB). As such, these cows were included to encompass the main factors: BT, OB or CB, and SF at milking, OSF or NSF, resulting in four treatments (OB-OSF, OB-NSF, CB-OSF, and CB-NSF) with 27 cows in each. The milking was performed using a portable machine (PLS-2/1; Sezer, Bursa, Türkiye) once daily (AM 05:00 to 08:00) at the stalls in the barns according to the manufacturer’s recommendations. In the OB-OSF and CB-OSF treatments, the TMR of 2 kg per cow was offered to the trough in front of stalls at milking.

#### Experiment II

Under the OB, farms offering different feeds during milking were enrolled in Experiment II. As explained in Experiment I, the cows in the farms enrolled in Experiment II were also observed. Then, 60 clinically healthy primiparous cows at approximately 90 days in milk were selected to assess the effect of offering different feeds at milking on the welfare assessment traits, milk quantity and quality, and profitability of the cows. Thus, five groups with 12 cows each were formed: i) no supplemental feed (CON), ii) offered a commercial concentrate (CC), iii) offered a CC + corn silage (CCS), iv), offered a CCS + alfalfa hay (CSA), or v) offered a CC + ryegrass silage (CRS) mixtures at milking., The commercial concentrate or the relevant PMR (2 kg per cow) of each treatment was offered to the troughs in front of stalls during milking performed using the portable machine once daily (AM 05:00 to 08:00) in the barns.

### Measurements and analyses

#### Welfare assessment

In both experiments, the evaluated welfare traits were observed and recorded in each cow accustomed to being offered feed at milking, consumed the TMR and the PMRs offered at milking, and habituated to the presence of the classifier. As previously explained [3], the welfare assessment traits were subjectively scored on a five-point scale (Table 1) by a single-trained classifier during the milking on test days. While milking temperament and body condition were scored during the morning milking, udder hygiene was scored immediately after milking. The welfare traits were assessed twice for two consecutive days at 7-day intervals during the observation of cows.

#### Milk yield and milk analyses

The milk collected in a tared bucket for each cow was weighed using an electronic scale to determine the MYM. Then, milk samples (approximately 50 mL) from the bucket belonging to each cow were individually placed in plastic milk tubes. These samples were transported to the laboratory at 4°C to assess the concentration of the milk components and physical traits [3]. The MYM of the cow was measured twice at 7-day intervals after 15 days of the observation duration.

All analyses were performed when the milk was at 30°C to 32°C. The chemical composition (milk fat, protein, lactose, and mineral percentages), the density (mg/mL), and the FP (°C) of the milk samples were analyzed using an automatic milk analyzer (Lactostar, Funke-Gerber, Germany). Also, milk SNF and fat-to-protein ratio (FPR) were calculated as all of the nutrients excluding fat and fat percentage/protein percentage, respectively. Milk EC (mS/cm) was measured using a FiveEasy Plus (Mettler Toledo, Switzerland) equipped with a conductometric sensor.

### Statistical analysis

All statistical analyses were conducted using the SPSS software program (version 21.0, SPSS Inc, Chicago, IL, USA). Before analysis, the normality and variance homogeneity of data from test-day records of welfare assessment and production (milk yield and quality) traits were screened using the Kolmogorov-Smirnov and Levene’s tests, respectively. In Experiment I, data on MYM and milk quality traits were analyzed using general linear model procedures with barn type and supplementary feeding as fixed effects and farm as a random effect (Y_ijk_ = μ+BT_i_+SF_j_+BT×SF_ij_+β_k_+e_ijkl_, in which Y_ijk_ is the quantitative response variable, μ is the overall mean, BT_i_ is the effect of barn type i, SF_j_ is the effect of supplementary feeding j, BT×SF_ij_ interaction effect ij, β_k_ is the effect of random (farm) k, and e_ijkl_ is the random error). In Experiment II, corresponding data were subjected to a one-way analysis of variance (Y_ij_ = μ+SF_i_+e_ijk_, in which Y_ij_ is the quantitative response variable, μ is the overall mean, SF_i_ is the effect of supplemental feed offered at milking i, and e_ijk_ is the random error) in a completely randomized design. Statistical significance was determined using Duncan’s multiple-range test and deemed significant when p<0.05. Data on welfare assessment traits in both experiments were analyzed using the Kruskal-Wallis H test, and the significant differences were determined with Dunn’s test at the p<0.05 level. All results were expressed as mean±standard error of the mean. Regression equations among welfare assessment traits and Pearson correlations among welfare assessment traits and between these traits and production traits were calculated, applying the linear regression and correlation procedure.

### Economic analyses

A partial budget analysis was conducted to provide a simple economic comparison of offering the TMR and the PMRs to buffalo cows at milking compared with un-supplemented animals as described by Windsor et al [23]. This approach was considered appropriate because it determines only the change in profitability resulting from changing a production practice [24]. The monetary value of the milk produced was calculated using the milk prices in markets based on milk quantity and quality. The increased milk quantity by TMR or the PMRs offered at milking compared with un-supplemented buffalo cows was considered extra revenue. Profit for the experimental groups that increased MYM was calculated by subtracting the feed and labor costs at milking from the monetary value of the extra milk quantity produced in the regarding group.

## RESULTS

### Experiment I

None of the cows scored higher than 3 and 4 points for body condition and udder hygiene, respectively. The barn type and offering supplemental feed at milking affected statistically the TS and UHS of the cows (Table 2). The TS (p = 0.021) and UHS (p<0.001) of the CB cows were higher (worse) than those of the OB cows. The TS (p = 0.021) and UHS (p< 0.001) of the OSF cows were lower (better) than those of the NSF cows. A barn type×supplementary feeding interaction was observed for the BCS, the MYM (p = 0.043), fat percentage (p = 0.014), and FPR (p<0.048). The OB-OSF cows had higher BCS than the OB-NSF, CB-OSF, and CB-NSF cows (p<0.05). The MYM of the OB-OSF cows was higher than those of the OB-NSF and CB-NSF cows (p<0.05). The milk fat percentage of the OB-OSF cows and the FPR of the CB-OSF cows were higher than those of other group cows (p< 0.05). The CB-NSF cows had milk with a lower fat percentage than the OB-NSF and CB-OSF cows (p<0.05). The OSF cows had higher milk protein (p = 0.007), lactose (p = 0.050), and SNF (p = 0.012) percentages and lower milk FP (p = 0.046) and EC (p = 0.011) values compared with the NSF cows.

**Figure 1:**
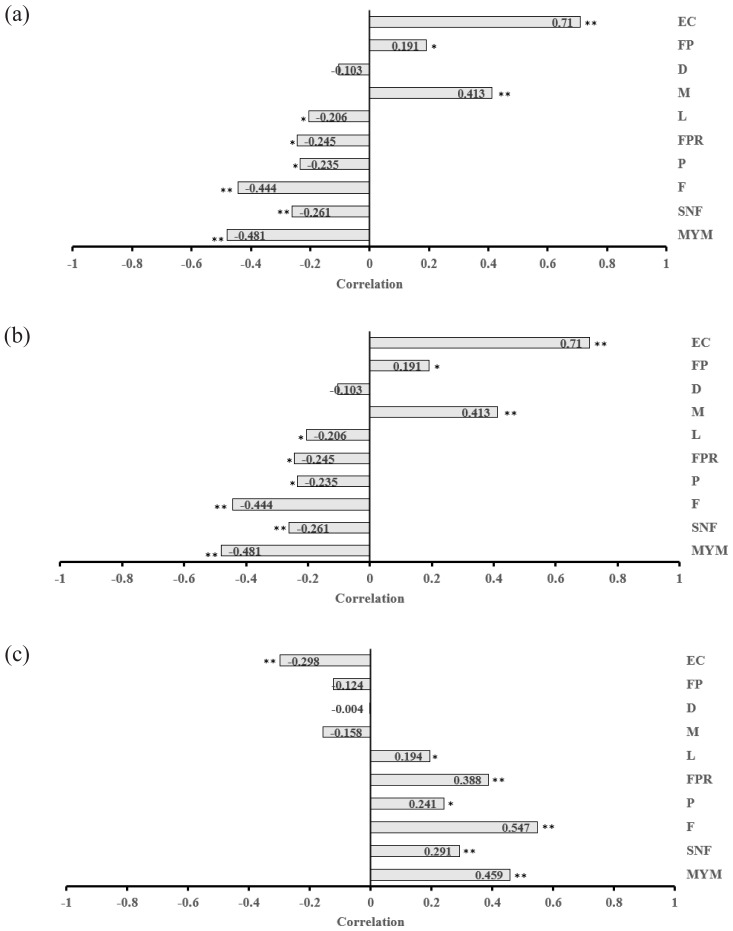
Direction, magnitude, correlation coefficients, and significance level of the correlations between (a) temperament score, (b) udder hygiene score, and (c) body condition score with milk yield and milk quality traits in buffalo cows. MYM, milk yield per milking; SNF, solids-non-fat; F, fat; P, protein; FPR, fat-to-protein ratio; L, lactose; M, minerals; D, density; FP, freezing point; EC, electrical conductivity. * p<0.05, ** p<0.01.

The buffalo cow TS was correlated with the UHS (r = 0.558) and BCS (r = −0.403), and UHS was correlated with BCS (r = −0.424) at milking (p<0.01; Table 3). The correlations of the welfare assessment traits with milk yield per milking and quality features are shown in Figure 1. The TS and UHS displayed a negative correlation with the milk yield per milking (p<0.01), the milk SNF (p<0.01), fat (p<0.01), and protein percentages (p<0.01), the FPR (p<0.01 for TS and p<0.05 for UHS), and a positive correlation with milk minerals percentage (p<0.05 for TS and p<0.01 for UHS) and the EC value (p<0.01). The positive correlation between the UHS and the FP was significant (p<0.05). The BCS correlated positively with the MYM (p<0.01), milk fat (p<0.01), SNF (p<0.01), protein (p<0.05), FPR (p<0.01), and lactose percentages (p<0.05) and negatively with the EC value (p< 0.01).

### Experiment II

The different feeds offered at milking affected the welfare assessment traits, milk yield per milking, milk chemical composition (except for protein and lactose percentages), and milk EC (Table 4). The cows offered PMR had lower TS, and their milk had lower EC than the CON and CC cows (p<0.05). The BCS was higher in cows offered CCS, CSA, and CRS feeds than in those offered CC feed (p<0.05). The CON and CC groups had lower BCS, MYM, fat percentages, and FPR than the other groups (p<0.05). Compared with the cows offered the CC feed, the CON and CCS cows produced milk with higher mineral percentages (p<0.05).

### Economic analyses

Supplemental feeds (TMR or PMRs) offered at milking may provide nutritional contributions, but their use did not offset feed requirements that differ from un-supplemented buffalo cows. Moreover, these practices may have further animal welfare, udder health, and productivity benefits. On the other hand, none of the farms reported any income losses associated with using supplemental feeds. Therefore, our partial budget analysis model did not include any reduced costs and returns foregone. Thus, the additional returns and extra costs were only derived from the increased milk production and the monetary values of supplemental feed and labor in each comparison set. The partial budget analysis identified that the OB-OSF group had a higher profit than the OB-NSF ($0.37) and CB-OSF ($0.11) groups, whereas the CB-OSF group showed a lower benefit than the CB-NSF and OB-NSF groups (Table 5). Compared with the CON group, the net profits of the CCS, CSA, and CRS groups were $0.21, $0.14, and $0.26, respectively.

## DISCUSSION

Our results indicated that i) the milk quantity and quality traits were enhanced by the supplemental feeds (TMR or PMRs) offered at milking, ii) there was an association between welfare assessment traits and also between the welfare assessment traits and some production traits, and iii) using TMR and the PMRs (especially CRS) at milking for OB buffaloes provided higher net profits. The increase in milk quantity and quality with additional feeding at milking can be explained by improved TS, UHS, and BCS, which affect these characteristics [3,14,15]. Our outcomes agree with the findings that buffalo cows classified as docile had significantly higher milk yield than those classified as nervous [3,8]. This indicates that docile and nervous buffalo cows under confinement react differently to environmental stimuli [1,8]. As such, buffalo cows’ milk quantity and quality respond to different aspects of the barn condition and feeding management at milking. In addition, these results suggest that offering TMR during milking promoted the welfare of primiparous buffaloes kept under intensive rearing conditions by encouraging them to adapt to milking systems [8]. In previous studies, this has been attributed to increased milk productivity [17], milking management [14,15], and the well-being of buffalo cows [3,17,21].

The applications in the present study improved, in general, the welfare assessment traits and the nutritional and physical-chemical properties of buffalo milk. Therefore, milking management applied herein may be a tool to overcome the challenges [6] of milking buffalo cows caused by intensive management techniques and the mechanization of daily farm activities [3,5]. As previously reported [10,21,25], the TMR or the PMRs at milking calmed Anatolian buffalo cows that were restless by machine milking [3]. The negative correlation between BCS with TS and UHS indicated a progressive decline in the BCS with increased welfare assessment scores. Indeed, we observed that nervous cows have a lower degree of cleanliness of the udder and hindquarters relative to calmer cows. Moreover, in the present study, TS and UHS negatively correlated with the MYM of primiparous buffalo cows. Contrary to our finding, Erdem et al [3] reported that TS positively correlated with the MYM in buffalo cows of lower parity. This contradiction may be because supplemental feeding at milking improved welfare assessment traits observed in the present study.

The mutual correlations among the welfare assessment traits suggest using the BCS as an auxiliary method for welfare assessment. Therefore, the observed BCS, lower than the ideal BCS of 3.0 to 3.25 [21,22], may indicate poor welfare status for buffalo cows in our study. Indeed, calmer cows had almost ideal BCS compared with nervous cows. Furthermore, in our study, the milk yield and components of cows with low TS and UHS vs high BCS were higher than their counterparts. The relationship between BCS and several milk components in the present study agrees with previous results from buffaloes [21] and dairy cattle [26]. However, scoring body condition may not be suitable for detecting differences in welfare status during early lactation [17] due to the intense mobilization of body fat until approximately 90 days of lactation [11].

Compared with the CB cows, higher welfare and productivity in OB cows may be related to the larger amounts of time the animals spend consuming feed, idling, and walking during the daytime in free-stall housing [5]. Furthermore, the reaction to an abrupt event by cows with free outdoor access is less intense than that of cows consistently kept in the CB [5,27]. Calmer buffalo cows may be better suited for operations wherein intensive handling is an aspect of the production system [1,2,5]. This elaboration suggests that cows that were unnecessarily vigilant during routine management practices or in the presence of any sudden stimulus had lower milk yield and quality traits [3,5,27].

Although cows with satisfactory BCS have stable blood metabolites for milk synthesis [9], UHS and TS may be necessary to improve milk quality and cow welfare because of the positive relationship between them, as noted by Erdem et al [3]. Moreover, while the BCS has positively correlated with these variables, the other two parameters are negatively correlated [3,9,17]. This may be related to promoted oxytocin release or decreased adrenaline levels regulating the milk release mechanism and milking efficiencies, such as milk flow rate, milking time, and milk yield of calmer buffalo cows, particularly primiparous cows [25]. Unfortunately, the present study did not investigate the hormonal responses of cows to barn conditions and feeding management at milking.

Changes in the milk density are a function of milk composition closely related to the milk SNF and fat percentages [16]. However, the milk density did not reflect the observed impacts of the studied applications on these two variables. The EC alone could allow the breeder to monitor the herd’s udder health in real-time and foresee the onset of problems [12,26]. The EC values in our study were lower than those reported for healthy (5.42 and 4.87 mS/cm), subclinical (6.16 and 5.37 mS/cm), and clinical (8.21 and 6.44 mS/cm) mastitis milk in buffaloes [28]. The positive relationships between UHS and milk EC and mineral percentages suggest that udder cleanliness is imperative for avoiding milk yield and quality losses, as stated in previous studies, and allowing the prediction of potential udder health problems [2]. In our study, milk FPR was close to or higher than those reported for primiparous buffalo cows [3,7].

Production and reproduction parameters are pivotal for the profitability and sustainability of dairy buffalo enterprises [29]. Milk lactose concentrations positively correlate with reproductive success and udder health [30]. The FPR, lactose percentage, and EC value are important indicators of temperament, udder health, fertility, and metabolic status [7,17]. In line with these reports, no metabolic disease, reproduction unsuccess, and udder health problems were observed in the present study. This knowledge may explain why changes in the TS, UHS, and BCS caused by the barn type and feeds at milking were reflected in milk’s chemical and physical properties [2,7]. Changes in the milk density are a function of milk composition closely related to the SNF and fat percentages of the milk [16]. The EC of milk, which is affected by the mineral content, is thought to influence the milk FP [2,12,30], which may explain the similar effect of offering supplemental feed at milking on the milk FP and EC values. Although lower milk production results from lower lactose, a vital osmotic component of milk content [26,31], the effects of the treatments investigated on the milk yield per milking were not reflected in the lactose percentage obtained in the present study. As such, our data can be a valuable reference for Anatolian buffaloes.

Offering a PMR at milking to primiparous cows showed a measurable positive impact on the welfare assessment traits, milk fat percentage, FPR, and EC value, as was found in Experiment I. The beneficial effect of the PMRs on welfare assessment traits may have resulted from regulating the direct or indirect impact of milking procedures and principles, such as approaching the animal, preparing the udder, attaching the milking unit, waiting for the milk flow, and detaching the milking unit [29,32]. This finding and data confirmed that buffaloes are very sensitive to the slightest change in milking routines, which is reflected in their milk yield [3,32]. Thus, these feeds may improve milk quantity and quality and labor efficiency by increasing the efficiency of the milking process, even though it is challenging to find literature concerning the different feeds offered at milking in free stalls [32].

The improvements in the MYM and quality traits for the buffalo cows offered a PMR at milking may be explained by two possible mechanisms. These treatments may have i) contributed to meeting their nutrient requirements [33] and ii) diminished the tendency of the cows to be unnecessarily vigilant against a sudden stimulus or an abrupt event during milking [3,5,27]. Our results did not fully confirm the first mechanism because the studied parameters were lower in CC than in all PMRs, despite the higher nutrient density and quality in the concentrate compared with the PMRs. This approach requires that not only the amount of feed offered to each cow but also the composition of the feed vary according to the different nutrient needs of the cows [33]. However, the CC treatment showed a favorable effect on the studied parameters by improving milking activity, indicating a contribution to nutritional requirements [33,34]. Hence, offering a PMR at milking may be an effective practice for maintaining the body fat reserves of the buffaloes, that is, the BCS [33,35].

The differences in the consumption time, structure, amount, and nutrient composition of any PMR indicate that the second mechanism was more descriptive than the first. The beneficial impact of the PRMs can be explained by focusing on feed consumption and extra time spent during milking [31,34] relative to the CC feed. Indeed, in our study, the CC feed was consumed in a shorter time because no choice was offered between feeds relative to any PRM (data not shown). Offering any PMR during machine milking could have encouraged voluntary milking and improved TS, UHS, and BCS associated with non-productive behavior in the OB conditions [27,36]. In our study, changes in the UHS and BCS of buffalo cows were reflected in the milk yield and fat percentage, as reported previously [21]. Although there was no difference in the consumption of feeds offered at milking, observed changes in the welfare assessment traits, suggest that there may be a preference for any PMR rather than the CC feed.

Our observations suggest that the increase in MYM, albeit from a low base milk yield level, as a result of the TMR and any PMR offered at milking was an advantage in terms of the economic return. Indeed, the partial budget analysis results indicated a strong incentive for the enterprise to include the TMR or PMR, except for CC feed at milking because the TMR and the PMRs, primarily CRS, provided a higher net benefit over their control counterparts. This provides additional evidence of the efficacy of milking technology in successfully enhancing productivity [29] and supplemental feeds to the generally inefficient buffalo production system [5,27], as recommended previously in Anatolian buffalo cows [3]. It should not be forgotten that the economic benefit depends on the stockperson-animal interaction and precision of the stockperson [5,27] during the provision (mixing and delivery) of TMR or PMR [23,32] to the cows.

## CONCLUSION

The factors in this study reflect distinct aspects of welfare assessments over milk yield per milking and milk quality traits of buffalo cows because i) there is a relationship between the well-being of animals and the selected milk features, ii) the temperament, body condition, and udder hygiene status of the buffaloes confined in semi-open free-stall barns was better than in closed-tied stall barns, iii) feeding any partial mixed ration during milking calmed the cows by reducing their interest in their surrounding during the milking process, and iv) Partial budget analysis suggested using TMR or partial mixed rations such as commercial concentrate + corn silage and commercial concentrate + ryegrass silage during milking, resulting in higher net profits for primiparous buffaloes confined in the semi-open free-stall barns. In conclusion, better health, milk yield, milk quality, and economy were seen in indoor primiparous buffalo cows offered TMR and/or roughage with concentrates at milking; however, the same benefits were not observed in the cows that provided TMR at milking in closed-tied stall barns. Further research should be focused on the influence of high parity on welfare assessment traits, milk yield, milk quality traits, and profitability in buffalo cows.

## Figures and Tables

**Table 1:** Subjective scores and meanings of the welfare assessment traits

Score	Welfare assessment trait		

	Temperament^1)^	Udder hygiene^2)^	Body condition^3)^
1	Very slow-very calm (docile)	Entirely clean	Emaciated
2	Slow-calm (slightly restless)	Clean	Thin
3	Normal (restless)	Dirty	Average
4	Sensitive-aggressive (nervous)	Very dirty	Fat
5	Very sensitive-very aggressive (aggressive)	Manure encrusted	Obese

1) Adapted from Antanaitis et al [17] and Shehar et al [18].

2) Adapted from Schreiner and Ruegg [19] and Reneau et al [20].

3) Adapted from Anitha et al [21] and Spengler Neff et al [22].

**Table 2:** The welfare assessment traits, milk yield per milking, and milk components and physical traits for primiparous buffalo cows offered or not supplementary feeding at milking at two barn types

Item	BT and SF^1)^				BT		SF		SEM	p-value^2)^		

	OB		CB									
				
	OSF	NSF	OSF	NSF	OB	CB	OSF	NSF		BT	SF	BT×SF
WAT^3)^												
Temperament score	1.38	2.96	2.03	2.96	2.01	2.51	1.71	2.80	0.119	0.021	<0.001	0.451
Udder hygiene score	1.50	1.85	1.96	2.67	1.67	2.33	1.73	2.27	0.092	<0.001	0.002	0.277
Body condition score	3.10^a^	2.66^b^	2.72^b^	2.58^b^	2.88	2.65	2.91	2.62	0.037	0.001	<0.001	0.022
MYM (kg)	3.51^a^	2.52^b^	2.83^ab^	2.35^b^	3.00	2.58	3.17	2.43	0.074	0.001	<0.001	0.043
Milk component												
Solids-non-fat (%)	11.21	10.25	10.75	10.17	10.72	10.42	10.95	10.21	0.148	0.307	0.012	0.473
Fat (%)	8.05^a^	7.31^b^	7.86^b^	6.55^c^	7.69	7.19	8.01	6.92	0.170	<0.001	<0.001	0.014
Protein (%)	4.86	4.50	4.49	4.48	4.66	4.68	4.69	4.50	0.069	0.497	0.007	0.711
Fat-to-protein ratio	1.64^b^	1.62^b^	1.76^a^	1.46^b^	1.67	1.53	1.71	1.54	0.042	<0.001	<0.001	0.048
Lactose (%)	5.09	4.90	5.06	4.85	4.99	4.95	5.07	4.87	0.051	0.693	0.050	0.935
Minerals (%)	0.64	0.69	0.66	0.65	0.65	0.66	0.65	0.67	0.010	0.728	0.276	0.217
Milk physical trait												
Density (mg/mL)	1.03	1.02	1.03	1.03	1.03	1.02	1.03	1.03	0.001	0.969	0.159	0.133
Freezing point (°C)	−0.76	−0.70	−0.73	−0.62	−0.73	−0.67	−0.74	−0.66	0.020	0.161	0.046	0.591
EC (mS/cm)	4.00	4.34	4.23	4.73	4.17	4.54	4.11	4.54	0.838	0.061	0.011	0.633

SEM, standard error of the mean; WAT, welfare assessment trait; MYM, milk yield per milking; EC, electrical conductivity.

1) Main factors = BT, barn type (OB, semi-open free-stall; CB, closed-tied-stall); SF, supplementary feeding (OSF, offered supplementary feeding at milking; NSF, not offered supplementary feeding at milking).

2) Probability of main effects of BT, SF, and interaction between BT and SF (BT×SF).

3) Temperament score, scale from 1 = very slow-very calm (docile) to 5 = very sensitive-very aggressive (aggressive); udder hygiene score, scale from 1 = entirely clean to 5 = manure encrusted, body condition score, scale from 1 = emaciated to 5 = obese.

a–c Mean values in the same row with different superscripts differ (p<0.05).

**Table 3:** Best fit regression equations, correlation coefficients, and significance level of the mutual correlations between temperament score (TS), udder hygiene score (UHS), and body condition score (BCS) observed at milking for primiparous buffalo cows

Model	UHS			TS		
	
	*β*_0_	*β*_1_	r	*β*_0_	*β*_1_	r
BCS	3.0982	−0.1651	−0.424^**^	3.0725	−0.1348	−0.403^**^
UHS				1.0266	0.4327	0.558^**^

*β*_0_, regression constant; *β*_1_, regression coefficient; *r*, correlation coefficients.

** p<0.01.

**Table 4:** The welfare assessment traits, milk yield per milking, and milk components and physical traits for primiparous buffalo cows offered commercial concentrate or partial mixed ration at milking at semi-open free-stall barn

Item	Treatment^1)^					SEM	p-value

	CON	CC	CCS	CSA	CRS		
WAT^2)^							
Temperament score	2.58^a^	2.75^a^	1.25^b^	1.41^b^	1.50^b^	0.140	>0.001
Udder hygiene score	2.41^a^	2.00^ab^	1.58^b^	1.66^b^	1.66^b^	0.112	0.034
Body condition score	2.68^b^	2.64^b^	3.06^a^	2.97^a^	3.04^a^	0.052	0.015
MYM (kg)	2.70^b^	2.34^b^	3.51^a^	3.35^a^	3.60^a^	0.102	<0.001
Milk component							
Solids-non-fat (%)	10.59	10.64	10.19	11.52	11.22	0.232	0.388
Fat (%)	6.86^c^	7.34^b^	9.23^a^	9.91^a^	9.70^a^	0.274	<0.001
Protein (%)	4.70	4.72	4.51	5.15	5.04	0.108	0.169
Fat-to-protein ratio	1.47^b^	1.56^b^	2.03^a^	1.94^a^	1.92^a^	0.071	<0.001
Lactose (%)	5.05	5.13	4.96	5.12	4.89	0.071	0.797
Minerals (%)	0.72^a^	0.60^b^	0.70^a^	0.67^ab^	0.65^ab^	0.011	0.016
Milk physical traits							
Density (mg/mL)	1.03	1.03	1.02	1.03	1.03	0.001	0.234
Freezing point (°C)	−0.74	−0.72	−0.74	−0.73	−0.78	0.011	0.510
EC (mS/cm)	4.38^a^	4.36^a^	3.98^c^	4.01^c^	4.10^b^	1.019	0.016

SEM, standard error of the mean; WAT, welfare assessment trait; MYM, milk yield per milking; EC, electrical conductivity.

1) CON, no supplemental feed (control) at milking; CC, offered commercial concentrate at milking; CCS, offered CC + corn silage at milking; CSA, offered CCS + alfalfa hay at milking; CRS, offered CC + ryegrass silage at milking.

2) Temperament score, scale from 1 = very slow-very calm (docile) to 5 = very sensitive-very aggressive (aggressive); udder hygiene score, scale from 1 = entirely clean to 5 = manure encrusted, body condition score, scale from 1 = emaciated to 5 = obese.

a–c Mean values in the same row with different superscripts differ (p<0.05).

**Table 5:** Partial budget analysis for the offered total mixed ration (Experiment I) and different partial mixed rations (Experiment II) to buffalo cows at milking

Partial budget	Experiment I				Experiment II			
	
	OB-OSF vs OB-NSF	CB-OSF vs CB-NSF	OB-OSF vs CB-OSF	CB-OSF vs OB-NSF	CC vs CON	CCS vs CON	CSA vs CON	CRS vs CON
Additional returns								
No. cows offered feed at milking (head)	27	27	27	27	12	12	12	12
Milk yield per milking (kg/d/cow)	3.51	2.83	3.51	2.83	2.34	3.51	3.35	3.60
Increased milk yield (kg/d/cow)^1)^	0.99	0.48	0.68	0.31	−0.36	0.81	0.65	0.90
Advantage of increase (%)	39.29	20.43	24.03	12.30	−13.33	30.00	24.07	33.33
Monetary value per kg of milk (USD)	1.06	1.06	1.06	1.06	1.06	1.06	1.06	1.06
Total additional returns (USD)	1.05	0.51	0.72	0.33	−0.38	0.86	0.69	0.95
Extra costs								
Amount of feed offered at milking (kg/cow)	2	2	2	2	2	2	2	2
Monetary value per kg of feed (USD)	0.23	0.23	0.23	0.23	0.18	0.22	0.20	0.27
Monetary value of feed offered (USD)	0.46	0.46	0.46	0.46	0.36	0.44	0.40	0.54
Days feeds deployed	1	1	1	1	1	1	1	1
Labor cost (USD×person hours)	0.15	0.15	0.15	0.15	0.15	0.15	0.15	0.15
Total extra costs (USD)	0.61	0.61	0.61	0.61	0.51	0.59	0.55	0.69
Net profit (USD)	0.37	−0.10	0.11	−0.28	−0.89	0.21	0.14	0.26

OB-OSF, offered supplemental feed at milking to buffalo cows confined in the semi-open free-stall barn; OB-NSF, not offered supplemental feed at milking to buffalo cows confined in the semi-open free-stall barn; CB-OSF, offered supplemental feed at milking to buffalo cows confined in the closed tied-stall barn; CB-NSF, not offered supplemental feed at milking to buffalo cows confined in the closed tied-stall barn; CON, not offered supplemental feed (control) at milking; CCS, offered CC + corn silage at milking; CSA, offered CCS + alfalfa hay at milking; CRS, offered CC + ryegrass silage at milking.

1) Values represent more produced milk quantity than the milk yield (kg/d/cow) in the OB-NSF (2.52), CB-NSF (2.35), CB-OSF (2.83), and CON (2.70) group in the relevant comparison set.
